# Cholinergic regulation of object recognition memory

**DOI:** 10.3389/fnbeh.2022.996089

**Published:** 2022-09-29

**Authors:** Kana Okada, Kouichi Hashimoto, Kazuto Kobayashi

**Affiliations:** ^1^Department of Neurophysiology, Graduate School of Biomedical and Health Sciences, Hiroshima University, Hiroshima, Japan; ^2^Department of Molecular Genetics, Institute of Biomedical Sciences, Fukushima Medical University School of Medicine, Fukushima, Japan

**Keywords:** basal forebrain, cholinergic system, hippocampus, muscarinic receptor, nicotinic receptor, perirhinal cortex

## Abstract

Object recognition memory refers to a basic memory mechanism to identify and recall various features of objects. This memory has been investigated by numerous studies in human, primates and rodents to elucidate the neuropsychological underpinnings in mammalian memory, as well as provide the diagnosis of dementia in some neurological diseases, such as Alzheimer’s disease and Parkinson’s disease. Since Alzheimer’s disease at the early stage is reported to be accompanied with cholinergic cell loss and impairment in recognition memory, the central cholinergic system has been studied to investigate the neural mechanism underlying recognition memory. Previous studies have suggested an important role of cholinergic neurons in the acquisition of some variants of object recognition memory in rodents. Cholinergic neurons in the medial septum and ventral diagonal band of Broca that project mainly to the hippocampus and parahippocampal area are related to recognition memory for object location. Cholinergic projections from the nucleus basalis magnocellularis innervating the entire cortex are associated with recognition memory for object identification. Especially, the brain regions that receive cholinergic projections, such as the perirhinal cortex and prefrontal cortex, are involved in recognition memory for object-in-place memory and object recency. In addition, experimental studies using rodent models for Alzheimer’s disease have reported that neurodegeneration within the central cholinergic system causes a deficit in object recognition memory. Elucidating how various types of object recognition memory are regulated by distinct cholinergic cell groups is necessary to clarify the neuronal mechanism for recognition memory and the development of therapeutic treatments for dementia.

## Introduction

Recognition memory is a simple type of declarative memory, defined as the ability to feel familiarity and to discriminate familiar items from unfamiliar ones (Mandler, 1980; Mackintosh, 1987; Squire, 1998). To evaluate recognition memory, spontaneous object recognition memory tasks are widely used in rodents (Ennaceur and Delacour, 1988; Dere et al., 2006; Aggleton and Nelson, 2020). *In such tasks*, animals are placed in an apparatus with objects, and they explore spontaneously. When object recognition memory is normally preserved, the time spent exploring novel objects is longer than that spent exploring familiar objects. This novelty preference is derived from the innate behavior of rodents to react to what was changed.

Previous studies have included experiments with numerous variants of the object recognition memory task to elucidate its neuronal mechanisms of recognition memory (Brown and Aggleton, 2001; Squire et al., 2007). Lesion studies showed that recognition memory for object location depends on the hippocampus and entorhinal cortex but not on the perirhinal cortex (Save et al., 1992; Parron et al., 2006). The suppression of the perirhinal cortex caused impairment in recognition memory for object identification, whereas the hippocampal lesion did not impair that memory (Save et al., 1992; Abe and Iwasaki, 2001; Brown et al., 2012). In addition, the medial temporal lobe is one of the brain regions that receive projections from cholinergic neurons in the basal forebrain (Bigl et al., 1982; Mesulam et al., 1983; Rye et al., 1984). Functional cooperation among the medial temporal lobe structures pivotally functions in several aspects of object recognition memory (Brown and Aggleton, 2001; Squire et al., 2007; Aggleton et al., 2012).

Clinical studies also suggest that the dysfunction of the basal forebrain cholinergic system causes impairment in recognition memory. Alzheimer’s disease is a severe memory disorder that is associated with a loss of cholinergic neurons in the forebrain, followed by neurodegeneration of a wide range of brain regions (Davies and Maloney, 1976; Pákáski and Kálmán, 2008; Schmitz and Zaborszky, 2021). The earliest sign of this disease is impairment in recognition of previously encountered stimuli (Ally, 2012). Cholinergic involvement in object recognition memory has been suggested by this clinical indication from Alzheimer’s disease. However, it remains unclear how the distinct cell groups in cholinergic systems are involved in the memory and interact with each other.

In the present review, we describe cholinergic regulation of object recognition memory, in which different cholinergic cell groups in the basal forebrain contribute to different aspects of memory. We also explain several behavioral factors that affect the performance in the memory task. Finally, we discuss the therapeutic possibility of cholinergic agents for correction of the impairment of object recognition memory seen in dementia.

## Central cholinergic system

**FIGURE 1:**
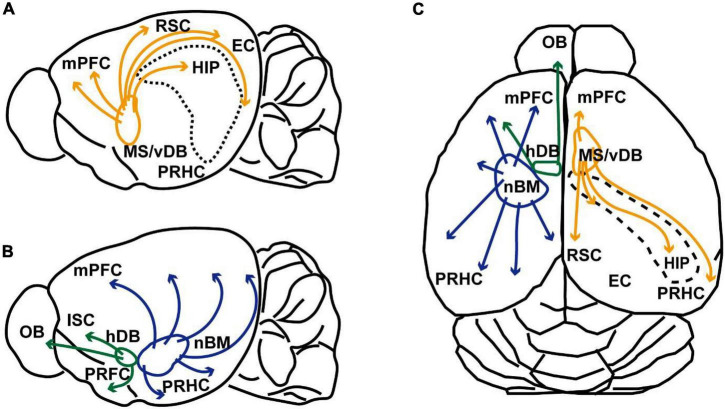
Schematic illustrations of cholinergic innervation from the basal forebrain of rodent. **(A)** Schematic sagittal view of the rodent brain illustrating cholinergic projection from the medial septum and ventral diagonal band of Broca (MS/vDB) to the medial prefrontal cortex (mPFC), retrosplenial cortex (RSC), entorhinal cortex (EC), hippocampus (HIP, and perirhinal/postrhinal cortices (PRHC). Cholinergic projections are indicated by orange lines. **(B)** Schematic sagittal view of the rodent brain showing cholinergic projection from the horizontal diagonal band of Broca (hDB) and nucleus basalis magnocellularis (nBM). Cholinergic neurons in the hDB innervates the olfactory bulb (OB), insular cortex (ISC) and piriform cortex (PRFC). Cholinergic neurons in the nBM project to the entire cortex including the mPFC and PRHC. Cholinergic modulations are indicated green and blue lines. Projections to the amygdala are omitted from the illustration. **(C)** Schematic dorsal view of the rodent cholinergic system. The right hemisphere shows cholinergic innervation from the MS/vDB. The left hemisphere indicates cholinergic projections from the hDB and nBM.

In the central nervous system, cholinergic neurons are composed of several distinct cell groups (Mesulam et al., 1983; Woolf et al., 1984; Woolf and Butcher, 1985; see Figure 1). Basal forebrain cholinergic neurons provide their projections to the entire neocortex and limbic cortex (Schmitz and Zaborszky, 2021). Cholinergic interneurons make local innervations within the striatum and neocortex (Mesulam et al., 1983; Zhou et al., 2002; von Engelhardt et al., 2007). In the cholinergic system, acetylcholine acts on nicotinic and muscarinic acetylcholine receptors, which are ionotropic and G protein-coupled metabotropic receptors, respectively (Levey et al., 1991; Alkondon and Albuquerque, 2004; Dani and Bertrand, 2007). These types of receptors are differentially distributed in the hippocampus, neocortex, and striatum in presynaptic and postsynaptic manners (Dannenberg et al., 2017; Obermayer et al., 2017).

Cholinergic neurons in the basal forebrain are divided into several groups; the medial septum (MS), ventral/horizontal diagonal band of Broca (vDB/hDB), and nucleus basalis magnocellularis or nucleus basalis of Meynert (nBM). The MS and vDB include cholinergic neurons projecting mainly to the hippocampus (the CA1-CA3, hilus, and dentate gyrus) and subiculum *via* the fornix. They also provide cholinergic innervations to the entorhinal, perirhinal, postrhinal, retrosplenial, infralimbic and prelimbic cortices (Gaykema et al., 1990; Gulyás et al., 1999; Kondo and Zaborszky, 2016). Cholinergic signaling in these projection areas has been assumed to occur both non-synaptically and synaptically (Vizi and Kiss, 1998; Zoli et al., 1999; Takács et al., 2018). Cholinergic neurons located in the hDB, innervate the main olfactory bulb, insular cortex and piriform cortex (Woolf et al., 1984; Záborszky et al., 1986). The caudal part of the basal forebrain cholinergic system consists of large cholinergic neurons in the nBM. This group includes cholinergic cells that are distributed throughout the ventral pallidum, magnocellular preoptic nucleus, nucleus basalis and substantia innominate. This cell group innervates the entire neocortex (isocortex) and amygdala (Mesulam et al., 1983; Eckenstein et al., 1988). They also innervate allocortical areas including the retrosplenial, entorhinal, and perirhinal cortices (Bigl et al., 1982; Woolf and Butcher, 1982, 1985; Rye et al., 1984; Woolf et al., 1984; Carlsen et al., 1985; Woolf, 1991).

## Various types of cholinergic system controlling object recognition memory

### Cholinergic projections from the medial septum and ventral diagonal band of Broca

**FIGURE 2:**
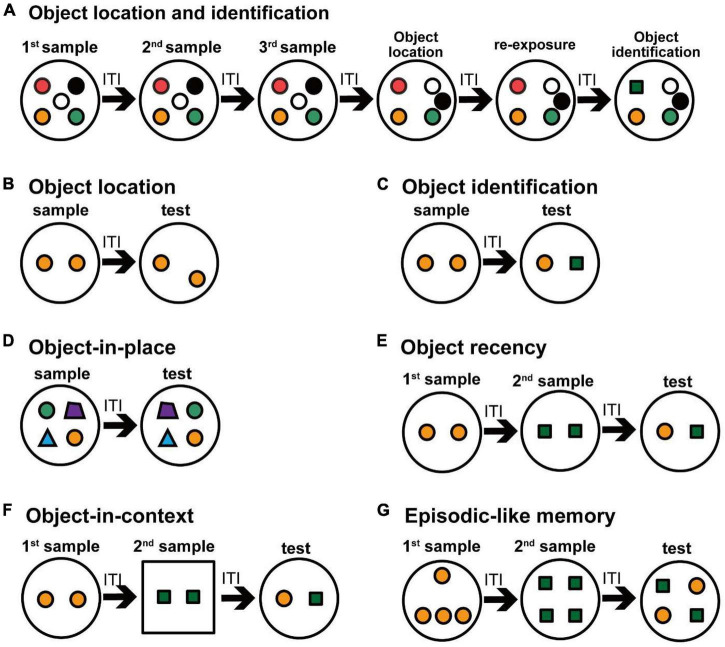
Schematic drawing of various object recognition tasks in rodents. Small colored circles and polygons indicate objects in an open field. Experimental protocols for evaluating the object recognition memory are shown. **(A)** The multiple-trial task evaluates the object recognition memory for the location and identification of the objects. In this task, successive six exposures are conducted with an ITI within 1 day. After three trials of sample exposure, two objects were relocated and an object location test is conducted. After re-exposure to the same arrangement objects in the object location test, a familiar object is replaced by a novel object in the object identification test. **(B–G)** One-trial tasks evaluate the object recognition memory, in which a sample trial and a test trial are conducted with an ITI on the same day, and some changes in the experimental conditions as for the objects are made in the test trial. In the object location task **(B)**, one of two objects is relocated in the test trial. In the object identification task **(C)**, one of two objects is replaced with another object in the test trial. In the object-in-place task **(D)**, two of four objects are relocated in the test trial. In the object recency task **(E)**, two objects in the first sample are exchanged by two other objects in the second sample, and then different objects in two samples are presented in the test trial. In the object-in-context task **(F)**, a set of objects in a context in the first sample are replaced with another set of objects in a different context in the second sample, and then different objects in two samples in the first context are presented in the test trial. In the episodic-like memory task **(G)**, four objects in the first samples are exchanged by four other objects in the second sample, and then the objects consisted of two objects from each sample are presented in the test trial.

Previous studies have revealed that cholinergic neurons in the MS/vDB are important in certain types of object recognition memory. A cholinergic lesion in the MS with 192 IgG-saporin decreases choline acetyltransferase activity in the hippocampus and frontal cortex, and impairs object location memory, but not object recognition memory (Cai et al., 2012). Selective cholinergic cell elimination in the MS/vDB by the immunotoxin-mediated cell targeting technique also impairs the object location memory in both multiple-trial and one-trial object recognition memory tasks (Okada et al., 2015; Figures 2A–C). One-trial recognition memory task simply consists of a sample trial and a test trial (Ennaceur and Delacour, 1988; Dere et al., 2006), whereas multiple-trial object recognition task is composed of some repeated sample and test trials (Poucet, 1989; Save et al., 1992; Okada et al., 2015). Amount of familiarization in the sample phase is reported to affect the performance in the test trials in object recognition memory (Albasser et al., 2009; Broadbent et al., 2010; Antunes and Biala, 2012). In contrast, another study reported that 192 IgG-saporin cholinergic lesions in the MS do not cause impairment of object location memory (Dashniani et al., 2015), although the difference in behavioral phenotypes may be because of their lesion sizes or subsections. For example, lesion of the MS left approximately 70% cholinergic neurons in the study of Dashniani et al. (2015), and their lesion size seems to be smaller than that in Okada et al. (2015). The injection sites of Dashniani et al. (2015) are located posterior in the MS to the sites of Cai et al. (2012). Injection sites of Okada et al. (2015) included a wide range of the MS/vDB along with the anteroposterior and mediolateral axes. The MS has a clear mediolateral topographical arrangement (Gaykema et al., 1990). The medial part of the MS projects to the dorsal hippocampus, the subiculum, and the lateral entorhinal cortex, whereas the lateral MS mainly projects to the ventral hippocampus, the subiculum, and the medial entorhinal cortex (Gaykema et al., 1990). In addition, neurons in the MS and rostral vDB mainly innervate the entire hippocampus, the subiculum and the entorhinal cortex, while neurons in the caudal vDB projects to the dorsal hippocampus, the dorsal subiculum and the lateral entorhinal cortex (Gaykema et al., 1990). The dorsal and ventral hippocampal structures are differently involved in mnemonic function (Hughes, 1965; Hock and Bunsey, 1998; Moser and Moser, 1998; Cassel et al., 2002). The medial and lateral entorhinal cortices are also differently implemented in the object recognition memory (Aggleton and Nelson, 2020). These anatomical and functional findings suggest that cholinergic neurons in subsections of the MS/vDB are differently involved in object location recognition memory or object-in-place recognition memory.

Cholinergic hippocampal activity is also reported to be important in object recognition memory (Aloisi et al., 1997; Giovannini et al., 2001; Stanley et al., 2012; Rashid and Ahmed, 2019). Neurochemical analysis shows that acetylcholine efflux in the hippocampus increases during spatial novelty and object exploration (Aloisi et al., 1997; Giovannini et al., 2001; Stanley et al., 2012). Pharmacological studies also indicate that cholinergic activity in the hippocampus and parahippocampal areas plays a role in novelty preference in several types of object recognition memory task. For example, the activity of muscarinic acetylcholine receptors in the hippocampus and entorhinal cortex is involved in the acquisition and retrieval of object location memory (Rashid and Ahmed, 2019). Acute activation of nicotinic receptors in the hippocampus or perirhinal cortex similarly enhances the acquisition of object recognition memory and object location memory, but not the retrieval of these memories (Melichercik et al., 2012). Local scopolamine infusion indicates that muscarinic activity in the hippocampus and perirhinal cortex is involved in short-term (90 min) object recognition memory, but muscarinic activity in the perirhinal cortex plays a role also in long-term (24 h) object recognition memory (Balderas et al., 2012). These results suggest that cholinergic hippocampal activity is involved in the performance of object location memory. It is unknown how cholinergic hippocampal activity modulates object recognition memory.

Cholinergic lesions of the MS with 192 IgG-saporin cause a deficit in object-in-context recognition memory, but not in episodic-like object recognition memory (Easton et al., 2011; Figures 2F,G). This impairment in object-in-context memory is suggested to be caused by failure in rapid updating of place cells when the object changes its environment. Indeed, MS cholinergic lesions with 192 IgG-saporin impair the development of new place cell representation in a novel context (Ikonen et al., 2002). Scopolamine infusion alters the firing properties of hippocampal place cells and grid cells in the entorhinal cortex (Brazhnik et al., 2004; Newman et al., 2014). Exploration in novel environments influences the firing properties of place cells and grid cells, suggesting that the increase of acetylcholine release in novel environment is related to alternation of firing patterns of these cells (Barry et al., 2012). Therefore, cholinergic activity in the hippocampus is strongly related to memory with salient spatial components.

### Cholinergic projections from the nucleus basalis of Meynert

Previous studies have revealed that cholinergic neurons in the nBM are important in a different type of object recognition memory from cholinergic neurons in the MS/vDB. A cholinergic lesion in the nBM by 192 IgG-saporin does not cause a novelty preference deficit in the object recognition memory test after 60-min delay (Savage et al., 2011). A selective cholinergic ablation in the nBM by the immunotoxin-mediated cell targeting technique also shows an intact novelty preference in the multiple-trial object recognition memory task, but it causes the impairment in one-trial object recognition memory after 3–30-min delays (Okada et al., 2015; Figures 2A,C).

Cholinergic neurons in the nBM project to the neocortex and amygdala, but also to the frontal, entorhinal, and perirhinal cortices (Woolf and Butcher, 1982, 1985; Rye et al., 1984; Woolf et al., 1984; Carlsen et al., 1985). Cholinergic transmission in the perirhinal cortex is reported to play a pivotal role in object recognition memory (Brown et al., 2012). Local infusion of methyllycaconitine or scopolamine in the perirhinal cortex impairs the acquisition of object recognition memory (Abe and Iwasaki, 2001; Winters and Bussey, 2005; Tinsley et al., 2011). Acute and pre-sample nicotinic receptor activation in the perirhinal cortex enhances novelty preference in the object recognition memory task (Melichercik et al., 2012). On the other hand, the cholinergic activity in the perirhinal cortex is not necessary for the retrieval of object recognition memory. Local scopolamine infusion into the perirhinal cortex does not affect object recognition memory during the test trial (Winters et al., 2006). Moreover, cholinergic activity in the perirhinal cortex is important in other variations of object recognition memory such as object-in-place and object recency memory (Brown et al., 2012; Figures 2D,E). Some studies have reported that the perirhinal cortex has no role in the object recognition memory in the absence of visual information (Winters and Reid, 2010; Albasser et al., 2013).

Acetylcholine in the medial prefrontal cortex is involved in novelty preference in the object recognition memory task (Esaki et al., 2021a,b). Nicotinic activation in the medial prefrontal cortex enhances the performance of object recognition memory (Esaki et al., 2021a,b). Scopolamine infusion into the medial prefrontal cortex impairs the acquisition of object-in-place recognition memory, but not the retrieval of the memory (Esaki et al., 2021a,b). This treatment also impairs the object recency memory (Barker and Warburton, 2011). Acetylcholine release in the prefrontal cortex is necessary for attention (Dalley et al., 2004; Nyberg, 2005; Bloem et al., 2014), suggesting that cortical cholinergic activity might be related to the acquisition of object recognition memory through its novelty-induced attention.

### Cholinergic projections from the horizontal diagonal band of Broca

There seems to be no report which indicates that cholinergic neurons of the hDB are related to object recognition memory, though cholinergic lesions in this area have been reported to increase depressive-like behaviors (Chen et al., 2021). The piriform cortex is reported to be important in processing odor-object recognition and integrating multisensory object information (Porada et al., 2019). On the other hand, there is the possibility that cholinergic projection to the perirhinal cortex is involved in object recognition memory *via* the hDB (Winters and Bussey, 2005). It is an issue to be addressed whether cholinergic projection from the hDB to the piriform and perirhinal cortices play a role in the processing of object recognition memory.

### Cholinergic interneurons

Striatal cholinergic interneurons are regarded as tonically active neurons (Kimura, 1986; Inokawa et al., 2010), and modulate striatal dopaminergic activity (Calabresi et al., 2000; Wang et al., 2006). Striatal cholinergic interneurons play a role in cognitive processes such as spatial working memory, reward-related learning (Kitabatake et al., 2003), habit learning (Packard and Knowlton, 2002; Aoki et al., 2018; Amaya and Smith, 2021), and behavioral flexibility (Ragozzino et al., 2009; Okada et al., 2014; Prado et al., 2017). Mice deficient in the vesicular acetylcholine transporter in the striatum have been reported to show impairment in short-term (15-min delay) object recognition memory (Palmer et al., 2016), indicating that cholinergic activity in the striatum is also relevant to the acquisition of object recognition memory. In contrast, there have been no reports to date on the role of cortical cholinergic interneurons in object recognition memory.

## Behavioral factors affecting object recognition memory

In the object recognition task, the experimenter uses the rodents’ inherent behavioral treat with their exploration and preference to the novelty, in order to evaluate the animals’ recognition memory. The rodents are able to react and re-explore the objects when the objects are altered with various properties, including material, size, and topographical arrangement or location (Cheal, 1978; Sutherland et al., 1982; Poucet et al., 1986; Thinus-Blanc et al., 1987; Ennaceur and Delacour, 1988; Save et al., 1992). This task does not require learning associated with any rules or any apparent reinforcements, but it is based on the inherent and spontaneous exploratory behavior toward novel or changed objects (Ennaceur and Delacour, 1988). Since the object recognition task uses the rodents’ spontaneous novelty preference that is measured by exploration to unfamiliar objects against more familiar objects, it is inevitable that the mentioned behavioral parameters of exploratory activity and attention would interfere the estimation of the object recognition memory (Antunes and Biala, 2012).

### Exploration in the open field

Evaluation of object recognition memory is based on the comparison between the explorations to unfamiliar and familiar objects in the test phase. When the animals show the lack or deficit of exploratory behavior itself, they are excluded from the data analysis of the experiments (Ennaceur and Delacour, 1988; Tinsley et al., 2011). Microdialysis studies in rodents have demonstrated that acetylcholine release in the cortex and hippocampus increases during exploration in a novel open field (Aloisi et al., 1997; Thiel et al., 1998; Giovannini et al., 2001). This increment of the acetylcholine levels gets shorter and smaller during re-exposure to the open field, suggesting that cholinergic activity is associated with exploration for novelty and declines according to habituation (Giovannini et al., 2001).

Cholinergic lesions in the basal forebrain by 192 IgG-saporin and systemic scopolamine administration do not alter rodents’ behavior in the open field (Psyrdellis et al., 2016; Dobryakova et al., 2018). In contrast, another report showed that cholinergic lesions led to hyperactivity in the open field (Waite et al., 1995). Systemic high-dose treatment (> 0.03 mg/kg) of scopolamine has been reported to impair locomotor activity (Klinkenberg and Blokland, 2010). These contradictory results suggest that the locomotor activity during the exploration appears to be altered by cholinergic dysfunction, depending on differences in the severity and location of the cholinergic lesion.

### Seeking novelty and attention

Animals show the novelty preference dependent on the integrity of their attention and memory in the test phase of object recognition memory (Silvers et al., 2007; Antunes and Biala, 2012). Several studies have shown that novelty signals during learning are associated with hippocampal or cortical acetylcholine transmission (Wilson and Rolls, 1990; Hasselmo, 1999; Ranganath and Rainer, 2003; Meeter et al., 2004; Barry et al., 2012). Acute nicotine administration improves attention and memory (Levin et al., 2006), and enhances novelty detection and subsequent recognition memory (Froeliger et al., 2009). Administration of scopolamine and mecamylamine revealed that nicotinic and muscarinic receptors are also important in attentional processing (Mirza and Stolerman, 1998, 2000; Klinkenberg and Blokland, 2010). A selective cholinergic lesion of the nBM or prefrontal cortex impairs attention and visual cue detection (McGaughy and Sarter, 1998; McGaughy et al., 2002; Chudasama et al., 2004; Klinkenberg and Blokland, 2010), suggesting that cholinergic modulation of attention and cue detection is mediated by the prefrontal cortex. The basal forebrain cholinergic system appears to regulate object recognition memory, at least partly, through attention.

## Impairments in object recognition memory in animal models for Alzheimer’s disease

Alzheimer’s disease is a progressive dementia. This disease is characterized by anterograde amnesia of short-term episodic memory, together with impairment in attention and spatial recognition at the early stage (Snowden et al., 2011). Impairment in recognition memory frequently occurs in patients at the prodromal stage of cognitive symptoms (Ally, 2012), and recognition memory deficit is one of biomarkers of Alzheimer’s disease (Russo et al., 2017; Goldstein et al., 2019). Cholinergic neurons in the basal forebrain are highly vulnerable to the effects of tauopathy in Alzheimer’s disease, and neuronal loss is generated in the basal forebrain area, but cholinergic cell loss is more severe in the nBM than in the MS/vDB (Geula et al., 2021). To mimic the key components associated with the early stage of Alzheimer’s disease, a selective elimination of cholinergic neurons in the rodent basal forebrain has been conducted for use as a valid model of Alzheimer’s disease at the early stage (Cutuli et al., 2009, 2013; Okada et al., 2015). These model mice show alterations in object recognition memory and object location memory (Cutuli et al., 2013; Okada et al., 2015).

Alzheimer’s disease is characterized by neuronal degeneration with the extracellular amyloid plaques and intracellular neurofibrillary tangles (Murphy and LeVine, 2010). The amyloid plaques are composed mainly of amyloid beta (Aβ) derived from the processing of amyloid precursor protein (APP), and neurofibrillary tangles are formed by hyper-phosphorylated tau protein (Zhang et al., 2006; Schmidt et al., 2009; De Strooper, 2010; Murphy and LeVine, 2010). Transgenic mouse models with some mutations in the genes encoding APP, presenilin, and tau have been reported to show deficits in object recognition memory (Dodart et al., 2000; Huang et al., 2006; Middei et al., 2006; Hillen et al., 2010; Zhang et al., 2012; Spilman et al., 2014; Grayson et al., 2015; Mehla et al., 2019). Moreover, object recognition memory was impaired by the intracerebroventricular injection of Aβ (Tsunekawa et al., 2008; Meunier et al., 2013). Deficits of object recognition memory in these model mice were rescued by the treatment of donepezil as an acetylcholinesterase inhibitor (Zhang et al., 2012), although there are contradictory results in other studies (Tsunekawa et al., 2008; Spilman et al., 2014). The impairments in object recognition memory and object location memory in themodels with cholinergic deletions have been reported to be recovered by treatment with donepezil or rivastigmine (Cutuli et al., 2013; Okada et al., 2015). Although it is still unknown how cholinergic activity is related to the neuropathology and cognitive decline, the object recognition memory task is a useful tool to study the mechanisms underlying the pathology of Alzheimer’s disease, and develop therapeutic treatments for dementia.

## Future aspects

This review revealed that distinct cholinergic cell groups in the basal forebrain are related to different types of object recognition memory. Cholinergic neurons in the MS/vDB innervating the hippocampal area are involved in object location recognition memory. Cholinergic neurons in the nBM projecting mainly to the entire neocortex have a role in object recognition memory. The perirhinal cortex plays an important role in object recognition memory, and receives cholinergic innervation from both the MS/vDB and nBM. Cholinergic activity in the prefrontal cortex is also necessary for object recognition memory. It is needed to determine which cholinergic cell groups projecting to the perirhinal or prefrontal cortex contribute to object recognition memory. Moreover, the contribution of cholinergic interneurons in the striatum and neocortex remains unknown. In addition, deficits in recognition memory are replicated in various rodent models of several neurological disorders, and the deficits can be rescued by cholinesterase inhibitors that activate cholinergic activity. It is unknown how the inhibitors work for the recovery of mnemonic dysfunctions caused by the neuronal degeneration in Alzheimer’s disease. Further experiments will help to explain how the distinct cholinergic neurons could control the cholinergic projection areas during the processes of object recognition memory. Elucidating the cholinergic regulation of object recognition memory will be useful for the development of therapeutic treatments for dementia.

## Author contributions

KO: writing—original draft, review, and editing and drawing illustrations. KH: writing—review and editing. KK: writing—original draft, review, and editing. All authors contributed to the article and approved the submitted version.
